# Precautionary Behaviors during the Second and Third Phases of the COVID-19 Pandemic: Comparative Study in the Latin American Population

**DOI:** 10.3390/ijerph18136882

**Published:** 2021-06-26

**Authors:** Rosa Martha Meda-Lara, Pedro Juárez-Rodríguez, Nayib Ester Carrasco-Tapias, Claudio Rodolfo Barrales-Díaz, Andrés Palomera-Chávez, Esteban González-Díaz, María del Carmen Llantá-Abreu, Lucia Lorenzana-Montenegro, Marta Herrero, Bernardo Moreno-Jiménez

**Affiliations:** 1 Centro Universitario de Ciencias de la Salud, Departamento de Psicología Básica, Universidad de Guadalajara, Guadalajara 44340, Mexico; rosa.meda@academicos.udg.mx (R.M.M.-L.); andres.palomera@academicos.udg.mx (A.P.-C.); 2 Centro Universitario de Ciencias de la Salud, Departamento de Biología Molecular y Genómica, Universidad de Guadalajara, Guadalajara 44340, Mexico; 3 Facultad de Psicología, Universidad Cooperativa de Colombia, Medellín 050012, Colombia; nayib.carrasco@ucc.edu.co; 4 Facultad de Salud, Universidad Central de Chile, Escuela de Psicología, Santiago 8320000, Chile; celapsa@gmail.com; 5 Centro Universitario de Ciencias de la Salud, Instituto de Patología Infecciosa y Experimental, Universidad de Guadalajara, Guadalajara 44340, Mexico; doc.glzdiaz@gmail.com; 6 Instituto Nacional de Psicooncología y Radiobiología, Sección Psicooncología y Trabajo Social, La Habana 10400, Cuba; mcllanta2014@gmail.com; 7 Asociación Guatemalteca de Psicología, 01010 Ciudad de Guatemala, Guatemala; llorenzana@ufm.edu; 8 Departamento de Psicología Social y del Desarrollo, Universidad de Deusto, 48007 Bilbao, Spain; marta.herrerolaz@gmail.com; 9 Facultad de Psicología, Universidad Autónoma de Madrid, Comunidad de Madrid, 28049 Madrid, Spain; bernardo.moreno@uam.es

**Keywords:** COVID-19, Latin America, precautionary behavior, confinement, concerns, medical symptoms

## Abstract

The population’s behavioral responses to containment and precautionary measures during the COVID-19 pandemic have played a fundamental role in controlling the contagion. A comparative analysis of precautionary behaviors in the region was carried out. A total of 1184 people from Mexico, Colombia, Chile, Cuba, and Guatemala participated through an online survey containing a questionnaire on sociodemographic factors, precautionary behaviors, information about COVID-19, concerns, maintenance of confinement, and medical symptoms associated with COVID-19. Cubans reported the highest scores for information about COVID-19. Colombians reported less frequent usage of precautionary measures (e.g., use of masks), but greater adherence to confinement recommendations in general, in contrast to the low levels of these behaviors in Guatemalans. Chileans reported greater pandemic-related concerns and the highest number of medical symptoms associated with COVID-19. These findings allow a partial characterization of the Latin American population’s responses during the second and third phases of the COVID-19 pandemic and highlight the importance of designing and managing public health policies according to the circumstances of each population when facing pandemics.

## 1. Introduction

Latin America as a whole has been severely affected by the COVID-19 pandemic due to the precariousness of health systems; insufficient health infrastructures; political problems that in some cases weaken the governance of the countries [1]; and the high prevalence of chronic diseases, poverty, and inequity [2]. Moreover, sociocultural elements have an impact on responses to pandemics and epidemics, particularly in developing countries [3].

In the face of the outbreak of a novel coronavirus (SARS-CoV-2), the causative agent of coronavirus disease 2019 (COVID-19), containment policies and precautionary measures have been adopted globally to halt its spread. Given that the success of such strategies relies on the behavioral responses of populations (i.e., adherence to measures such as home confinement, social distancing, and use of masks) [4], it becomes necessary to identify the distinctive features of the behavioral responses of different countries in order to design interventions appropriate for each sociocultural context.

In this sense, information about COVID-19 has proven very useful in promoting precautionary behaviors [5], as well as maintaining them [6]. According to Pfattheicher et al., when empathy toward the most vulnerable people is induced, adherence to precautionary measures is promoted [7]. However, information overload, false reports, and rumors on social media, all facilitated by the speed of their dissemination through the Internet, can be harmful to mental health [8] and can produce fear [9] and maladaptive behaviors [10]. Therefore, it is not surprising that information about transmission modes, symptoms, precautionary behaviors, and personal hygiene are the main topics in the media.

People get information not only from social media and news reports, but also from other forms of popular culture, such as television, movies, and fictional novels, among others. This information is a key factor in the rejection of quarantines, isolation, and other means of controlling pandemic outbreaks [11]. In this sense, mandatory confinement has been one of the main interventions established by authorities to prevent the spread of coronavirus, given its mode of transmission even by asymptomatic carriers [12]. However, social distancing and quarantining have negative effects on the mental health of the population, causing fear and anxiety [13], moderate to severe depression, emotional distress [14], post-traumatic stress symptoms, confusion, and anger [15].

The similarity of conditions among Latin American populations has often been mentioned; however, the evolution of the pandemic differs by a multitude of variables in the social, cultural, and economic context of each country, as shown by early reports [16]. In Mexico, the first confirmed case was registered on 27 February 2020. The Mexican government implemented a series of measures to prevent and control the spread of infection within the country, which included branding a National Journey of Healthy Distance, and an Aid Plan for Disasters (DN-III-E Plan) protocolized and implemented primarily by the Mexican Secretariat of National Defense. Certain economic activities were suspended, massive congregations of people were restricted, and the domiciliary confinement of the general population was extended [17]. According to the Mexican government, as of 10 June, the total number of confirmed cases was 124,184, and the number of deaths was 15,357 [18].

In Chile, the first confirmed case occurred on 3 March 2020. The actions taken by the government were as follows: the declaration of a constitutional state of emergency, the establishment of close contacts, mandatory and dynamic quarantines, night curfew, sanitary residences, unification of the public and private health system, increase in the number of beds in the ICU, medical insurance for catastrophic care, cash subsidies for vulnerable population, and police permission to circulate [19]. The total number of confirmed cases by 10 June was 148,496, and the number of deaths was 2475 [19]. Guatemala has been considered vulnerable to the pandemic due to the low number of mechanical ventilators in the country [20]; however, there is little accurate information regarding the number of cases, deaths, tests performed, and their distribution [21]. According to official figures, the first confirmed case occurred on 12 March 2020, the total number of confirmed cases by 11 June was 13,624, and the number of deaths was 503 [22].

The case of Colombia has been considered an innovative way of dealing with the pandemic through measures such as family allowances during quarantine, telemedicine and home care provision, and task-shifting strategies for healthcare workers to provide basic health services, among others [21]. Colombia addressed the pandemic early with containment measures with policies such as closures of ports, universities, and schools; quarantine for migrants; and social isolation. The first confirmed case occurred on 6 March 2020, the number of deaths by 8 June was 400, and the total number of confirmed cases by 12 June was 29,998 [23]. Cuba has unique aspects in the region such as a well-organized primary healthcare system, a high number of physicians per million inhabitants, and experience in rapid evacuations in emergency situations as well as during epidemics, which contributed to an adequate and effective response through mass surveillance, contact tracing, and the use of isolation centers [24]. The first confirmed case occurred on 11 March 2020, the total number of confirmed cases by 24 June was 2321, and the number of deaths was 85 [24].

Therefore, the present study aimed to perform a comparative analysis of the precautionary behaviors, levels of information, concerns, medical symptoms, and maintenance of confinement in several Latin American countries, which will contribute to the subsequent development and optimization of interventions that consider the characteristics of each country studied so as to improve the behaviors of the population when facing pandemics.

## 2. Materials and Methods

Given the restrictions imposed over the face-to face interaction during the data-collection period, the questionnaires and the informed consent letter were converted into an electronic format in the SurveyMonkey platform, and the e-survey was built upon the CHEcklist for Reporting Results of Internet E-Surveys (CHERRIES) [25]. A snowball sampling strategy was used, allowing for the gradual incorporation of informants from various Latin American countries who were asked to share the survey among their usual contacts [26]. The data were collected from 4 May to 11 June 2020. A total of 1184 participant responses from Mexico, Colombia, Cuba, Chile, and Guatemala were collected, with more than 100 responses per country. A survey completion rate of 77% was obtained.

By default, multiple responses are turned off in SurveyMonkey platform on the basis of cookies; moreover, to ensure single responses, we eliminated duplicate database entries having the same IP address, and first entry was kept for analysis. Completion of all items was enforced using JAVAScript (i.e., displaying an alert before the questionnaire can be submitted). Respondents were able to review and change their answers before submitting the survey. Responses were automatically captured by SurveyMonkey. No incentives were offered for participation.

A study by Wang et al. [27] on the impact of COVID-19 in the Chinese population, as well as other studies on the influence of pandemic outbreaks on populations’ behaviors [28,29,30], were analyzed as part of the present study, which comprised two sections. The first section collected data on the sociodemographic aspects most commonly used to describe a population, such as sex, age, education, country of origin, occupation, family relationships, children and elderly dependents, and family size.

The second section included a survey organized into five sections:

- Information about COVID-19—five questions to establish the levels of information about COVID-19, satisfaction with public information, and primary sources of information (e. g. “public information about COVID-19 has been”, “my satisfaction with the information received from experts has been”). A 5-point Likert scale was used to answer, from “none” = 1 to “a lot” = 5. Additionally, when asked about their main source of information (“my main source of information has been”), participants could answer “internet”, “television”, “radio”, “family members”, “other, which one?”
- Concerns related to COVID-19—nine questions about the levels of concern caused by issues such as medical and institutional resources and training, the possibility of becoming ill or having family members become ill, the possibility of dying, and the loss of financial resources (e.g., “I am concerned about hospital’s resources to take care of the ill”, “I am concerned about the likelihood of getting infected during the current outbreak”, “I am concerned about the likelihood of being hospitalized”). A 5-point Likert scale was used to answer, from “none” = 1 to “a lot” = 5.
- Precautionary measures during COVID-19—seven questions on following the recommendations of authorities and experts, the use of masks, hand washing, and maintenance of social distancing, among others (e.g., “covering my mouth when coughing and sneezing”, “avoiding sharing utensils (e. g. fork)”, “washing my hands with soap and water”, “wearing mask regardless of the presence or absence of symptoms”). A 5-point Likert scale was used to answer, from “never” = 1 to “always” = 5.
- Maintenance of confinement—four questions on compliance with the home confinement established by the authorities (e.g., “maintenance of confinement”, “time spent in home confinement”, “I have had to go out to work and interact with other people”, “maintenance of activities”). A 5-point Likert scale was used to answer, from “I have been away from home all the time” = 1 to “I have not been out at all” = 5.
- Medical symptoms—participants were asked about the presence of COVID-19-related symptoms in the 14 days prior to the survey, including: fever, cold, headache, muscle pain, cough, shortness of breath, dizziness, rhinitis, and sore throat. Presence of chronic illness, medical consultation in the past 14 days, quarantine in the past 14 days, and indirect contact with and individual with confirmed COVID-19 infection were collected as data.

Questions related to precautionary behaviors, concerns, maintenance of confinement, and information about COVID-19 were translated and adapted from the survey questions used in a study conducted during the pandemic outbreak in China [27]. A pilot run including 50 participants was conducted before the commencement of the research to assess the comprehensibility of all items and the usability, as well as the technical functionality of the electronic survey.

### 2.1. Ethical Considerations

The research project was evaluated and approved by the Ethics and Research Committee of the University Center for Health Science of the Universidad de Guadalajara (Mexico), with folio number CI-01520. All participants included in the study voluntarily provided their informed consent after reading the purposes of the study. Data are stored in a locked and password-protected computer under principal investigator’s safekeeping to maintain confidentiality.

### 2.2. Statistical Analysis

The data obtained were analyzed with the SPSS v.23.0 statistical package (IBM Corp, Armonk, NY, USA). The significance level was set at α < 0.05. Descriptive statistics were used when addressing the sociodemographic characteristics, information, concerns, precautionary behaviors, maintenance of home confinement, and medical symptoms. In addition, analysis of variance (ANOVA) was performed to compare the study variables by country for the continuous variables, and by chi-squared (χ^2^) contrasts for the categorical variables. In ANOVAs, η^2^_p_ was included to estimate the effect size, defined as small (η^2^_p_ > 0.10), medium (η^2^_p_ > 0.25), and large (η^2^_p_ > 0.40) effect [31]. In χ^2^ tests, Cramer’s V was calculated as indicator of effect size, defined as small (V > 0.10), medium (V > 0.30), and large (V > 0.50) effect [31].

## 3. Results

### 3.1. Development of the COVID-19 Pandemic in the five Latin American Countries during the Data Collection Period

**Figure 1:**
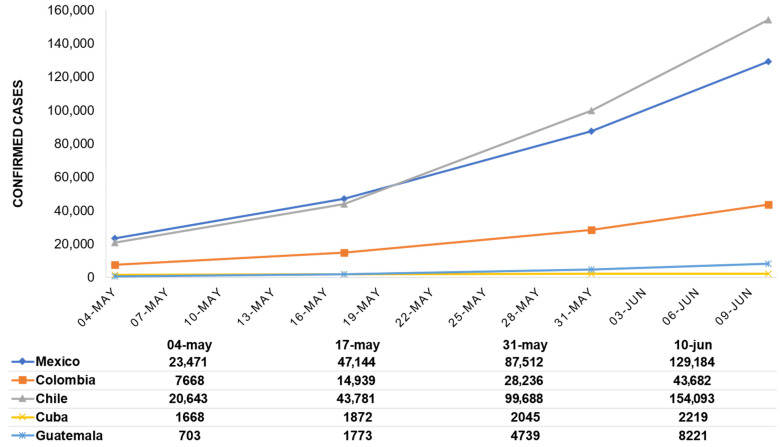
Development of the COVID-19 pandemic in the number of confirmed cases by country. Data obtained from the WHO coronavirus (COVID-19) Dashboard [32].

**Figure 2:**
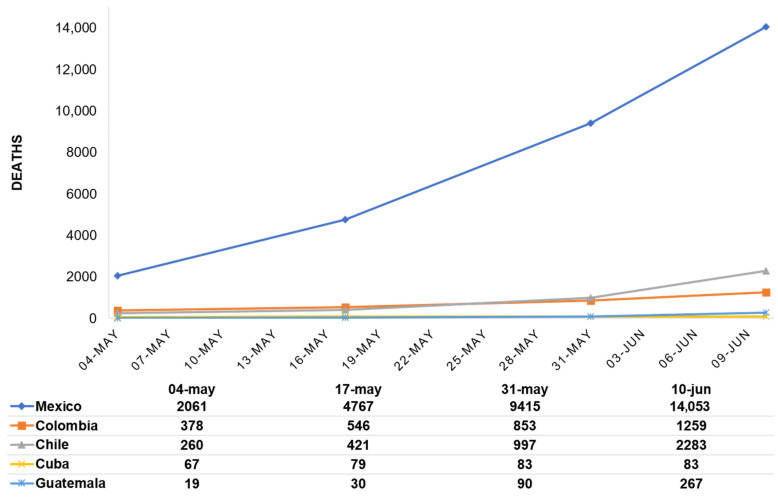
Development of the COVID-19 pandemic in the number of deaths by country. Data obtained from the WHO coronavirus (COVID-19) Dashboard [32].

Since the first reported case of infection in each country the number of confirmed cases and deaths have continued to escalate. Chile has the highest number of confirmed cases (Figure 1), however, Mexico is the country with the highest number of deaths (Figure 2).

### 3.2. Description of the Populations

Table 1 shows the composition of the sample. As regards the proportion of participants per country, approximately 60% were from Mexico; however, there were more than 100 participants from each country. Mean age of the sample was 38.78 years (SD = 13.81), with an age range of 18 to 83 years. An ANOVA was performed to compare age by country. Significant differences were found—the Colombian sample was younger than the rest of the countries; there were no differences between the rest of the countries.

Categorical sociodemographic variables were compared by country using Pearson’s χ^2^. Most of the participants were women (70.78%) in all countries, with similar gender ratios between Mexico, Colombia, Chile, and Guatemala and between Mexico, Chile, and Cuba. Colombia and Guatemala had significantly higher proportions of women. In terms of relationships Cuba, Guatemala, and Chile showed the same proportion of people who were single or in a stable relationship as Mexico. Colombia had a significantly higher percentage of single participants and a lower percentage of stable relationships. Moreover, all countries showed a similar proportion of participants with children under 16 years of age, but Colombia had a significantly lower percentage of people with children over 16 years of age and the highest percentage of people without children compared to the rest of the countries.

At least 60% of the participants from all countries reported having family members over 60 years of age. Similar proportions were found between all countries; nevertheless, Cubans reported the lowest number of family members over 60 years of age. Regarding the educational level, most of the countries were characterized by having participants with a high academic attainment. In all countries, bachelor’s degrees were most frequent, except in Colombia, where a basic education was predominant and significantly more frequent than in the rest of the countries. Similarly, the occupations with the highest percentage were students and teachers in the general sample. When comparing this variable between countries, in Guatemala and Colombia, there was a significantly higher percentage of administrative and technical occupations.

### 3.3. Information about COVID-19

The level of information on COVID-19 was compared by country using ANOVA, and significant differences were found in all of the variables, with the Cuban sample reporting the highest levels of information, information searching, public information, and satisfaction with the information provided by the experts in their country. The rest of the countries displayed a moderate level of information and searching. Regarding satisfaction with the information received from experts and authorities, almost all of the countries reported low satisfaction, except for the Cuban sample, with a moderate level. Finally, more than 80% indicated that their main source of information was the Internet, which was common in all countries (see Table 2).

### 3.4. COVID-19-Related Concerns

Table 3 presents the ANOVA on COVID-19-related concerns. The results indicate statistically significant differences in all variables. Cubans indicated a low level of concern regarding the availability of sanitary resources to face the pandemic (i.e., “I am concerned that doctors have enough resources to diagnose” and “I am concerned that hospitals have enough resources to care for the hospitalized”), the possible loss of economic resources, and being unemployed, in comparison to the rest of the countries studied. With respect to concerns directly linked to COVID-19 infection, Guatemalans indicated that they were less concerned about the probability of dying from COVID-19 or the possibility of infecting family members compared to the rest of the countries studied. Regarding “Concern about the possibility of infection of family members,” the responses were similar among the countries, with a slight difference between Guatemala and Chile (with Guatemalans being less concerned).

### 3.5. Precautionary Behaviors

Table 4 presents the ANOVA of COVID-19 precautionary behaviors by country. Statistically significant differences were found in all behaviors. In the Colombian sample, behaviors such as covering the mouth when coughing or sneezing, avoiding sharing utensils, and washing hands with soap and water were reported to a lower extent than in the rest of the countries studied. On the contrary, the Mexican sample indicated less use of masks and gloves. Likewise, Cuba was the country that reported the highest use of masks, while Colombia and Chile reported the highest use of gloves. Participants from Guatemala reported the highest frequency of hand washing after handling contaminated objects. Chileans and Guatemalans had higher scores in the maintenance of protective distance compared to the rest of the countries studied.

### 3.6. Maintenance of Confinement

Table 5 presents the ANOVA of confinement-related behaviors during the COVID-19 pandemic by country. Statistically significant differences were found in all behaviors. In all cases, the Colombian sample showed the highest scores, while in the cases of “maintenance of confinement,” “confinement time,” and “restriction of exits,” the Guatemalans indicated the lowest levels. With respect to “restriction of interaction at work,” the Cuban population obtained the lowest score.

### 3.7. Comparative Analysis by Medical Symptoms

Comparative analysis was performed using χ^2^ between medical symptoms by country. Statistically significant differences by rate were found in relation to the total number of medical symptoms presented by the respondents at the time of the survey. As shown in Table 6, nearly 90% of the Cuban sample reported having no symptoms, followed by Guatemala with almost 70%. More than 50% of the participants in the Chilean sample reported having between one and three symptoms, followed by Colombia with 48%. Moreover, the Chilean sample indicated the most symptoms (<10%), reporting having between four and seven symptoms. In relation to having chronic diseases (e.g., hypertension, diabetes, cancer, and respiratory problems) or having been in quarantine for COVID-19 in the last 14 days, the Cuban and Guatemalan samples indicated higher frequencies than the rest of the countries; specifically, they showed between 7% and 15% higher frequencies of suffering from chronic diseases than the rest of the countries, where this frequency was close to 20%. Cuba and Guatemala also reported practically no cases of quarantine, while in the rest of the countries, quarantine was observed in around 5% of the cases. Mexicans and Colombians reported similar frequencies of having been in quarantine for COVID-19 in the last 14 days. Furthermore, the Cuban sample reported the lowest frequency of having visited a doctor in the last 14 days. Regarding indirect contact with persons with confirmed COVID-19, the contrast showed significant global differences, with 100% of respondents in Cuba reporting that they had not been in indirect contact with any person with COVID-19, while in the rest of the countries, there were cases.

## 4. Discussion

The worldwide impact of the COVID-19 pandemic on health and the economy is evident; however, developing countries are especially vulnerable due to the higher proportion of rural areas, larger populations, limitations in the health field, poverty levels, and inequity in access to information [2,4].

In Latin America, authors such as Almeida Espinosa and Sarmiento Ardila have described the different ramifications of the disease in Colombia [33]. Ríos González and Palacios have done the same in Paraguay [34]. Dagnino et al. highlighted the effects of the pandemic in the Chilean population [35]. Other authors have analyzed the context and the initial response of several Latin American countries to the pandemic [16,20,36]. The data collected during the second and third phases of COVID-19 from the populations in Mexico, Colombia, Chile, Guatemala, and Cuba present a series of characteristics that help to understand the development of the pandemic and the responses to it in the Latin American population.

According to morbidity and mortality data, there was a marked increase in infections in the five Latin American countries during the data collection period (Figure 1 and Figure 2). Interestingly, Cuba and Guatemala reported the lowest numbers of confirmed cases and deaths among the countries studied: on the one hand, Cuba has a prepared health system and experience in emergency situations [24], and on the other hand, Guatemala has what is considered a vulnerable health system [20]. Moreover, despite having similar numbers of confirmed cases, Mexico and Chile differ significantly in the number of deaths due to COVID-19. These results demonstrate the need to continue investigating the elements that lead to the adoption or rejection of precautionary measures, since in all the countries analyzed, governments imposed lockdowns, restricted circulation and created working groups to coordinate efforts, however, the data shows an upward trend in infections.

The results of the sample composition describe relatively homogeneous characteristics such as a predominance of the female gender, mostly with children, with family members aged 60 years and with a high educational attainment. However, at the same time, the results highlight some differences, for instance, the younger sample from Colombia, as well as its higher proportion of people without a couple and its lower academic attainment The Internet is the main source of information used by people in the populations studied (about 80%), consistent with the findings in Asian populations [27,37,38]. Differences were identified among the Latin American countries studied. The Cuban sample was characterized by a higher level of and searching for information, more information, and greater satisfaction with the information provided by experts and authorities in their country. The rest of the countries (i.e., Mexico, Colombia, Chile, and Guatemala) reported a moderate level of information and low satisfaction with the information received by experts and authorities in their countries. Mass media communications and trust in the government can affect dysfunctional safety behavior such as panic buying [39,40]. Therefore, and in line with the recommendations made by other research groups [41], it would be advisable for the authorities to take advantage of the internet as a preferred channel for communication and possible intervention while trying not to saturate the population with information, and taking special care with the way information is presented, as it has been reported that this can be counterproductive [8,9,42].

Despite fears and concerns, beliefs and customs related to health and disease have hardly benefitted the development of precautionary and responsible behaviors in the face of epidemics [43]. Preventive behaviors in the face of pandemics are the result of information, education, and fears [44], and therefore their practice is a manifestation of the immediate perception of disease risk [45]. In the population surveyed, diversity was found in the precautionary behaviors in relation to COVID-19. Colombians were characterized as taking fewer measures, such as covering their mouths when coughing or sneezing, avoiding sharing utensils, and washing hands with soap and water, in relation to the rest of the countries. Mexicans showed a low level of use of masks and gloves, in contrast to Cubans, who reported a higher use of masks. Therefore, the most common recommended precautionary behaviors are generally followed, except for the use of gloves and masks; similar results were found in Saudi Arabia, with low levels of using masks (16.9%) [38], in contrast to the high levels of use of masks in the Chinese population (59.8%), as described by Wang et al. [27]. The study by Muto et al. showed that the Japanese population has high levels of all precautionary measures, even without a government mandate [46].

Regarding the maintenance of containment measures, the sample of Colombians showed greater adherence to the recommendations, time of confinement, and exit restrictions in contrast to the low levels of these behaviors in Guatemalans. In this regard, Idrovo commented that the social conditions of the countries in the region, which include low educational levels and high levels of poverty, unemployment, and informality, have led to the relaxation of restraint measures, with a view to reactivating the economy [47]. On the contrary, empathy has been highlighted as a relevant factor in the adherence to care and precautionary measures during this pandemic [7].

Although the effect of a pandemic depends on its extent and severity, it also depends on the resources available to a nation [16,48]; the results of this study comparing the medical and economic concerns, as well as the medical symptoms, by country denote the differences between populations within the Latin American context and reaffirm the importance of designing precautionary measures according to the circumstances of each population.

In general, the Cuban sample showed better adaptation to the COVID-19 pandemic, although they had the highest percentage of participants with chronic illnesses, such as hypertension, diabetes, or cancer. Cubans showed the lowest levels of concern about having health resources, and less concern about losing economic resources or becoming unemployed in relation to the rest of the countries. At the level of COVID-19 symptoms, almost 90% of the Cuban sample reported not having any symptoms in the 14 days before answering the survey. This may be due to the effectiveness of their recognized strategy against COVID-19, given their extensive and organized primary care system, high number of physicians per million in the population, and their experience [36,48,49]. On the contrary, Mexicans and Guatemalans exhibited less concern about getting sick, being hospitalized, dying from COVID-19, or older relatives becoming infected.

There are several limitations to this study. First, online studies can be subject to bias, for instance, the non-representative nature of the internet population; however, given the limited resources and following government measures adopted during the second and third phases of the COVID-19 pandemic in which face to face interactions was restricted and home confinement extended, an online platform was used. Efforts were made to minimize bias by following the CHERRIES statement for online studies [25]. In this sense, we suggest future follow-up studies and/or online panel research methods to represent populations more accurately.

Second, the snowball sampling strategy used to collect information allowed rapid access to the population in five Latin American countries during an international public health emergency in which other strategies could have been unsafe, but at the same time, restricted data representativeness that can limit the reach of our conclusions, but not invalidate them. In this sense, our study informs about tendencies in responses and is inserted in the global effort to show the pandemic repercussions on a regional level.

Third, precautionary behaviors involve multiple determinants that demonstrate substantial interindividual variability; in this regard, our study could be used as a reference for future studies aimed at delving deeper into those determinants beyond the country of origin.

Several current publications address the precautionary behavior in different countries [4,27,50,51], but very few studies analyze the issue in Latin America with empirical data. Notwithstanding the limitations, this study provides valuable information regarding the precautionary behaviors, information about COVID-19, concerns, maintenance of confinement, and COVID-19-related symptomatology derived from respondents across five Latin American countries, emphasizing the importance of developing programs for infectious disease prevention as well as precautionary behaviors and health lifestyle promotion. As Mackenzie et al. indicated, COVID-19 is the third zoonotic epidemic in the last two decades [52].

The most pressing need is to research the negative biopsychosocial impacts of the COVID-19 pandemic to facilitate immediate and longer-term recovery, also in relation to behavior and adherence to precautionary measures [53]. Results obtained in the present study may help funders and policymakers make informed decisions about future research priorities to best meet the needs of the countries and in the development of tailored public health policies and communications that facilitate compliance with precautionary measures.

## 5. Conclusions

In the five countries studied, there are similarities in the implementation of campaigns aimed at preventing the contagion of the populations, for instance, the declaration of quarantines starting in March, promotion of physical distancing, closure of educational centers and recreational centers, closure of non-essential economic activities, and mandatory use of masks, among the most important ones. Our findings allow a partial characterization of the Latin American population’s responses during the second and third phases of the COVID-19 pandemic, highlighting the importance of designing and managing public health policies according to the circumstances of each population when facing pandemics. Future studies should address the public and health promotion policies that countries have implemented to control the pandemic, as well as programs aimed at modifying risk behaviors and promoting health from an interdisciplinary perspective.

## Figures and Tables

**Table 1:** Sociodemographic data by country.

Variable	Mexico (*n* = 680)	Colombia (*n* = 149)	Chile (*n* = 128)	Cuba (*n* = 106)	Guatemala (*n* = 120)	χ^2^	V
	*n* (%)	*n* (%)	*n* (%)	*n* (%)	*n* (%)		
Gender							
Women	474 (69.70)	120 (80.50)	91 (71.10)	61 (57.00)	92 (76.70)	19.06 ***	0.127
Men	206 (30.30)	29 (19.50)	37 (28.90)	46 (43.00)	28 (23.30)		
Relationships							
In a stable relationship	433 (63.68)	73 (49.00)	75 (58.60)	62 (58.50)	74 (61.67)	23.80 **	0.100
In an unstable relationship	33 (4.85)	7 (4.70)	11 (8.60)	8 (7.50)	0 (0.00)		
Single	214 (31.47)	69 (46.3)	42 (32.80)	36 (34.00)	46 (38.33)		
Family members older than 60 years							
Yes	537 (79.00)	112 (75.20)	96 (75.00)	66 (61.70)	99 (82.50)	18.19 ***	0.124
No	143 (21.00)	37 (24.80)	32 (25.00)	41 (38.30)	21 (17.50)		
Children							
Has children under 16 years	192 (28.20)	38 (26.00)	42 (32.80)	28 (26.20)	45 (37.50)	68.70 ***	0.170
Has children over 16 years	162 (23.80)	9 (6.00)	23 (18.00)	43 (40.20)	39 (32.50)		
No children	326 (48.00)	102 (68.00)	63 (49.20)	36 (33.60)	36 (30.00)		
Educational level							
Basic education	77 (11.30)	80 (53.69)	25 (21.09)	54 (50.50)	31 (25.80)	332.45 ***	0.265
Bachelor´s degree	320 (47.10)	32 (22.82)	72 (56.25)	52 (48.60)	51 (42.50)		
Master´s degree or higher	283 (41.60)	33 (23.49)	28 (22.66)	1 (0.90)	38 (31.70)		
Occupation							
Student	134 (19.71)	85 (57.05)	50 (39.06)	9 (8.49)	11 (9.17)	423.48 ***	0.314
Professor	179 (26.32)	23 (15.44)	18 (14.06)	9 (8.49)	11 (9.17)		
Administrative employee	104 (15.30)	27 (18.12)	14 (10.94)	14 (13.21)	23 (19.16)		
Other occupations	234 (34.41)	11 (7.38)	40 (31.25)	7 (6.60)	35 (29.17)		
Unemployed	29 (4.26)	3 (2.01)	6 (4.69)	0 (0.00)	0 (0.00)		
No answer	0 (0.00)	0 (0.00)	0 (0.00)	67 (63.21)	40 (33.33)		

Note: 63.21% of Cubans and a 33.33% of Guatemalans did not provide information about their occupation. Basic education includes elementary school and high school. ** *p* ≤ 0.01, *** *p* ≤ 0.001.

**Table 2:** ANOVA of the levels of information about COVID-19 for country.

Variable	Mexico	Colombia	Chile	Cuba	Guatemala	F	η^2^_p_
	M ± DT	M ± DT	M ± DT	M ± DT	M ± DT		
Level of information	2.85 ± 0.90	2.68 ± 0.93	2.99 ± 0.95	3.84 ± 0.83	2.88 ± 0.89	31.21 **	0.096
Search of information	2.50 ± 1.09	2.52 ± 1.08	2.56 ± 1.24	3.38 ± 1.07	2.46 ± 1.13	15.44 **	0.050
Public information	2.72 ± 1.00	2.87 ± 0.90	2.71 ± 1.10	4.01 ± 0.92	2.59 ± 1.15	40.63 **	0.121
Satisfaction with the information	1.43 ± 0.99	1.41 ± 0.77	1.55 ± 1.04	2.69 ± 1.11	1.53 ± 1.10	6.66 **	0.022

Note: ** *p* ≤ 0.001.

**Table 3:** ANOVA of COVID-19-related concerns by country.

Variable	Mexico	Colombia	Chile	Cuba	Guatemala	F	η^2^_p_
	M ± DT	M ± DT	M ± DT	M ± DT	M ± DT		
Concerns about doctor´s resources to diagnose	3.17 ± 0.91	2.87 ± 1.04	3.34 ± 0.71	2.33 ± 1.21	3.09 ± 0.95	22.63 **	0.071
Concerns about hospital´s resources to take care of the ill	3.34 ± 0.86	3.13 ± 1.01	3.54 ± 0.60	2.41 ± 1.25	3.46 ± 0.76	31.07 **	0.095
Likelihood of getting infected during the current outbreak	2.20 ± 0.93	2.17 ± 1.01	2.48 ± 0.93	2.42 ± 1.21	2.23 ± 0.92	3.30 *	0.011
Likelihood of being hospitalized	1.98 ± 0.96	2.13 ± 1.01	2.23 ± 1.05	2.28 ± 1.22	2.21 ± 1.06	3.95 *	0.013
Likelihood of die because of COVID-19 infection	1.81 ± 0.95	1.99 ± 1.03	2.00 ± 1.09	2.24 ± 1.28	1.80 ± 0.96	5.26 **	0.018
Concerns about other family members getting COVID-19 infection	2.80 ± 1.10	2.89 ± 1.03	3.09 ± 0.97	2.95 ± 1.14	2.75 ± 1.11	2.43 *	0.008
Concerns about older family members getting COVID-19 infection	2.93 ± 1.12	2.93 ± 1.10	3.28 ± 0.94	2.83 ± 1.24	2.89 ± 1.09	6.46 *	0.011
Concerns about losing important economic resources	2.20 ± 1.17	2.25 ± 1.16	2.16 ± 1.20	1.61 ± 1.13	2.21 ± 1.13	6.46 **	0.021
Concerns about losing the job	1.86 ± 1.17	1.98 ± 1.22	2.01 ± 1.21	1.37 ± 0.93	1.84 ± 1.10	5.57 **	0.019

Note: * *p* ≤ 0.05; ** *p* ≤ 0.001.

**Table 4:** ANOVA of precautionary behaviors by country.

Variable	Mexico	Colombia	Chile	Cuba	Guatemala	F	η^2^_p_
	M ± DT	M ± DT	M ± DT	M ± DT	M ± DT		
Covering my mouth when coughing and sneezing	3.64 ± 0.73	3.36 ± 0.88	3.48 ± 0.83	3.56 ± 0.84	3.59 ± 0.76	4.55 **	0.015
Avoiding sharing of utensils (e.g., fork)	3.19 ± 1.21	2.66 ± 1.45	3.14 ± 1.18	3.27 ± 1.31	3.31 ± 1.12	6.58 **	0.022
Washing my hands with soap and water	3.50 ± 0.83	3.21 ± 1.00	3.47 ± 0.76	3.42 ± 1.01	3.55 ± 0.84	3.93 *	0.013
Wearing mask regardless of the presence or absence of symptoms	1.83 ± 1.47	2.85 ± 1.31	3.27 ± 1.05	3.70 ± 0.73	3.43 ± 1.01	93.18 **	0.241
Wearing protection gloves	0.75 ± 1.11	1.62 ± 1.43	1.48 ± 1.38	0.82 ± 1.44	1.08 ± 1.33	21.29 **	0.068
Washing my hands immediately after touching contaminated objects	3.44 ± 0.96	3.41 ± 0.95	3.56 ± 0.78	3.34 ± 1.04	3.68 ± 0.71	2.69 *	0.009
Keeping distance from other people	3.33 ± 0.91	3.38 ± 0.86	3.62 ± 0.64	3.42 ± 0.88	3.61 ± 0.73	4.89 **	0.016

Note: * *p* ≤ 0.05; ** *p* ≤ 0.001.

**Table 5:** ANOVA of maintenance of containment measures during COVID-19 by country.

Variable	Mexico	Colombia	Chile	Cuba	Guatemala	F	η^2^_p_
	M ± DT	M ± DT	M ± DT	M ± DT	M ± DT		
Maintenance of confinement	2.86 ± 0.62	3.02 ± 0.62	2.88 ± 0.66	2.97 ± 0.93	2.73 ± 0.73	3.92 *	0.013
Confinement time	3.39 ± 0.64	3.68 ± 0.67	3.45 ± 0.67	3.26 ± 0.78	2.97 ± 0.95	18.17 **	0.058
Restriction of interaction at work	2.93 ± 0.85	3.15 ± 0.83	2.99 ± 0.87	2.24 ± 0.93	2.86 ± 0.84	19.42 **	0.062
Restriction of exits	3.13 ± 0.56	3.30 ± 0.55	3.17 ± 0.57	3.21 ± 0.63	3.03 ± 0.72	4.22 *	0.014

Note: * *p* ≤ 0.05; ** *p* ≤ 0.001.

**Table 6:** Medical symptoms during COVID-19 pandemic by country.

Variable	Mexico *n* (%)	Colombia *n* (%)	Chile *n* (%)	Cuba *n* (%)	Guatemala *n* (%)	χ^2^	V
Medical symptoms							
None	385 (56.62)	69 (46.31)	48 (37.50)	95 (88.79)	83 (69.17)	82.31 **	0.186
1–3	262 (38.53)	71 (47.65)	66 (51.56)	12 (11.21)	32 (26.67)		
4–7	33 (4.85)	9 (6.04)	14 (10.94)	0 (0.00)	5 (4.16)		
Chronic illness							
Yes	141 (20.74)	29 (19.46)	37 (28.91)	40 (37.38)	27 (22.50)	214.35 **	0.425
No	539 (79.26)	120 (80.54)	91 (71.19)	67 (62.62)	93 (77.50)		
Medical consultation in the past 14 days							
Yes	93 (13.68)	16 (10.74)	14 (10.94)	3 (2.80)	11 (9.16)	11.81 *	0.100
No	587 (86.32)	133 (89.26)	114 (89.16)	104 (97.20)	109 (90.84)		
Quarantine in the past 14 days							
Yes	136 (20.00)	31 (20.81)	21 (16.41)	0 (0.00)	5 (4.26)	42.82 **	0.190
No	544 (80.00)	118 (79.29)	107 (83.59)	107 (100.00)	115 (95.84)		
Indirect contact with an individual with confirmed COVID-19 infection.							
Yes	33 (4.85)	2 (1.34)	8 (6.25)	0 (0.00)	6 (5.00)	10.09 *	0.092
No	647 (95.15)	147 (98.76)	120 (93.75)	107 (100.00)	114 (95.00)		

Note: * *p* ≤ 0.05; ** *p* ≤ 0.001.

## Data Availability

The data presented in this study are available on request from the corresponding author. The data are not publicly available due to the protection of personal data that could compromise the privacy of research participants.
